# In-hospital airway management of COVID-19 patients

**DOI:** 10.1186/s13054-020-03018-x

**Published:** 2020-06-05

**Authors:** Elise H. Sullivan, Lauren E. Gibson, Lorenzo Berra, Marvin G. Chang, Edward A. Bittner

**Affiliations:** 1 Department of Anesthesiology, Pain Medicine, and Critical Care, The Massachusetts General Hospital, Harvard Medical School, Boston, MA USA; 2 Division of Cardiac Anesthesia and Critical Care, Department of Anesthesia, Critical Care and Pain Medicine, Massachusetts General Hospital, Boston, USA

**Keywords:** Emergency airway management, Airway management, In-hospital airway management, COVID-19, coronavirus, Intubation, COVID-19 airway management, Coronavirus airway management

## Abstract

Those involved in the airway management of COVID-19 patients are particularly at risk. Here, we describe a practical, stepwise protocol for safe in-hospital airway management in patients with suspected or confirmed COVID-19 infection.

As the number of patients infected with coronavirus disease 2019 (COVID-19) has grown exponentially, the number of patients who require intubation may be greater as non-invasive ventilation is utilized less frequently due to increased risk for aerosolization of viral particles. Rapid viral transmission within hospitals has been documented and remains a major concern [1–4]. In Italy, healthcare workers comprised 9% of total COVID-19 cases [5], with respiratory therapists, intubating providers, and bedside nurses being at highest risk [6]. At our 999-bed hospital with over 350 concomitant COVID-19-positive inpatients, 508 employees have tested positive for COVID-19 out of 3466 tested. The emerging data presents an alarming and unprecedented need for preparation. We anticipate that hospital resources will be taxed with increasing numbers of COVID-19-infected patients in the coming weeks. Healthcare workers will increasingly be at risk of exposure, especially those involved in airway management. It is imperative to have a practical, stepwise protocol for safe hospitalization and airway management in patients with suspected or confirmed COVID-19 infection.

## High-risk aerosolizing procedures

Several commonly performed medical procedures are likely to aerosolize patient sputum and thus significantly increase the risk of exposing healthcare workers to respiratory pathogens. Tracheal intubation, positive pressure ventilation with bilevel positive airway pressure (BiPAP) or continuous positive airway pressure (CPAP), high-flow nasal cannula (HFNC), bronchoscopy, and nebulizer treatments should be regarded as high risk for COVID-19 transmission [7]. These procedures should be employed with precautions in patients with COVID-19 infection. A systematic review of healthcare-associated transmission of betacoronavirus during the SARS epidemic showed that tracheal intubation, non-invasive positive pressure ventilation, tracheostomy, and manual ventilation prior to intubation were all associated with a significantly increased risk of transmission to healthcare workers [8]. Although the other procedures listed do not yet have data showing increased transmission, we use risk of aerosolization of patient sputum as a proxy marker for risk of transmission. We recommend treating all potentially aerosolizing procedures as carrying an elevated risk of exposure to healthcare workers. Additionally, any contact with mucous membranes confers a high risk of viral infection. Both the aerosolization of infectious particles and contact with mucus membranes make airway management especially hazardous.

## Avoid emergent intubations: precaution takes time

Given the increased risk of exposure to healthcare providers, all efforts should be made to avoid emergently intubating patients with COVID-19. Firstly, hand hygiene is a crucial step in protection from viral spread. All healthcare providers involved must have adequate time for application of airborne precaution personal protective equipment (PPE) prior to airway management. Airborne PPE for COVID-19 consists of eye protection, a fit-tested respirator such as an N95 mask, a fluid resistant gown, and gloves. Intubators may consider double gloving such that one layer of gloves may be discarded after securing the airway, before handling any other equipment. *A powered air-purifying respirator (PAPR) provides high caliber protection and may be worn if available.*

We recommend creating a portable PPE kit to ensure protection for high-risk procedures in a global atmosphere of shortage. Although PPE should be available throughout the hospital, it is not always realistic to guarantee rapidly available PPE across varying clinical locations. This PPE kit should include gloves, gowns, head covers, shoe covers, face shields and/or protective eyewear, and N95 respirators. After use, all PPE should be removed and discarded in the anteroom. With patient rooms that lack an anteroom, all PPE except the N95 mask should be removed prior to exiting the patient’s room. The mask should be removed and securely discarded once the provider has exited the patient’s shared airspace. It is important that each team member observes donning and doffing of other team members in order to prevent contamination.

## N95 versus PAPR for respiratory PPE: pros, cons, and practicality

When comparing the N95 mask to a PAPR for protection of the healthcare provider performing airway management, the pros and cons of each respirator must be considered. N95 masks filter approximately 95% of aerosol particles (< 5 μm) and droplets (5–50 μm), are more readily available and faster to don, do not require a power source, and allow use of a stethoscope. In addition, they are less expensive and more readily available. N95 masks do not prevent contamination of face and neck and can be rendered ineffective by poor fit, as due to weight changes or presence of facial hair [9, 10]. Decontamination of face and neck should be considered after airway management with an N95 mask.

PAPRs offer some advantages when compared to N95 masks. PAPRs do not require individualized fit testing to ensure adequate functional protection. The face and neck are protected by a full hood, and they may be better tolerated for prolonged periods of care, since they do not force the provider to breathe against a high-resistance filter. The disadvantages include requiring connection to a power source such as a battery, impaired communication due to the noise of positive airflow and filter, inability to use a stethoscope, and the risk of contamination for anyone disposing of or re-processing the PAPR filter [9]. We recommend use of N95 masks for their fast application, availability, and more consistent staff familiarity.

These airborne precautions are also recommended for asymptomatic COVID-19-positive patients. Case reports have shown efficient spread of COVID-19 from patients exhibiting no viral symptoms, as well as from those who have recovered from the infection yet continue to shed the virus. Of note, case reports are emerging of surgical patients infecting multiple healthcare workers before any evidence of symptoms [11].

## Respiratory rounds: close interval respiratory evaluation

An institution’s airway response team (ART) may consider rounding on patients with suspected or confirmed COVID-19 infection for close evaluation of clinical trajectory. This approach may be especially beneficial in circumstances in which the number of infected patients exceeds the number of patients able to be accommodated in an ICU. As the pandemic grows, we recommend that each institution create a designated patient unit in order to isolate COVID-19 patients from other inpatients and allow for centralized monitoring and dissemination of information specific to COVID-19 care.

All patients with confirmed or suspected COVID-19 should have close interval evaluation to preempt acute respiratory failure rather than emergently react to it. Early admission should be considered for those patients who (1) have progressive respiratory symptoms due to severity of disease, (2) are elderly or have multiple comorbidities and thus diminished physiologic reserve, or (3) are anticipated to have difficult airways due to anatomic characteristics or clinical history. Although fundamental to all patient care, it is extremely important that the care team has an explicit discussion about the patient’s code status with the patient or family. Inappropriately intubating a patient with COVID-19 infection due to ignorance of the patient’s “Do Not Intubate” (DNI) status is not only a serious adverse event, it also exposes healthcare workers to unwarranted risk.

## The COVID-19 airway response team (ART)

It is important that physicians with specialized training in airway management are involved in the care of this patient population. Early trainees or physicians who do not have extensive experience with airway management should not intubate, except in dire circumstances without alternatives. Inexperience may increase the risk of improper adherence to peri-intubation precautions and viral transmission. In addition, inexperience is likely to result in more intubation attempts with associated complications and greater COVID-19 exposure of the clinicians performing the procedure. Having more than one team member with advanced airway training is advisable as preparation for potential difficult airways. The literature shows increased first pass intubation success and a reduction of complications of emergency tracheal intubation when an experienced intubator performs the procedure [12]. In our institution, the COVID-19 ART consists of a critical care-trained anesthesiologist, a critical care fellow, and a trauma surgeon as backup in the event that a surgical airway is needed. Each institution must devise a team that properly reflects resources available. This team may include experienced nurse anesthetists (CRNA) or anesthesiologist assistants (AA). Identifying the responding team in advance creates an organized system for rapid intervention and facilitates practices to protect the healthcare workers involved. The team member with the most airway experience should be the one to intubate; in our model, it is the critical care-trained anesthesiologist. All members of the COVID-19 ART must be educated in proper application of PPE.

For providers with advanced age, significant comorbidities, or immunocompromised status or who are currently pregnant, we have given the option to opt out of participating in the COVID-19 ART.

## Communication and consultation with the airway team

**Fig. 1:**
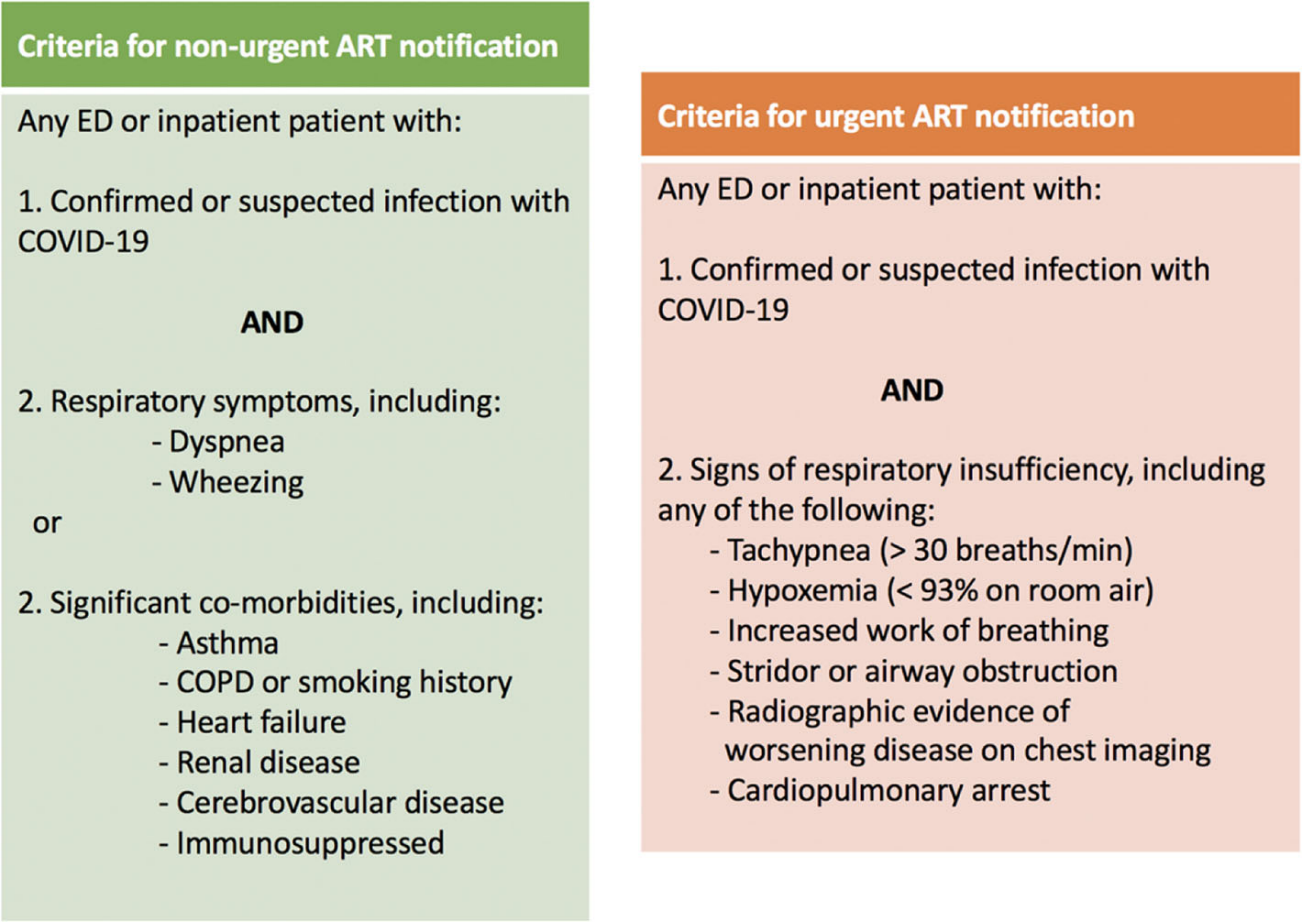
Suggested criteria for notification of a COVID-19 airway response team (ART)

**Fig. 2:**
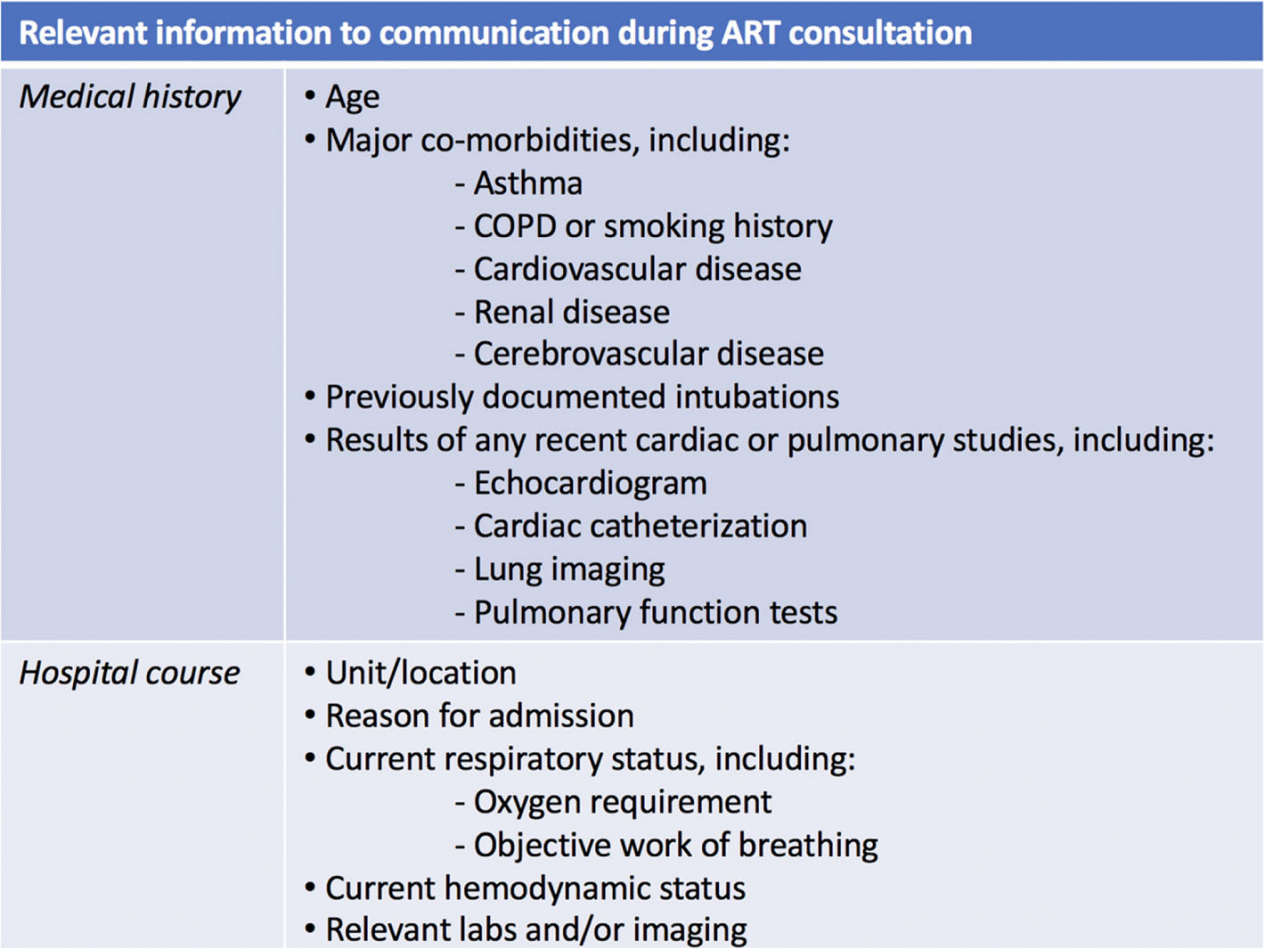
Relevant information to communicate during airway response team (ART) consultation

The ART should be notified of all confirmed or presumptive positive cases of COVID-19 in the hospital with evidence of respiratory distress; specifically, escalating oxygen requirements, stable oxygen support of greater than 4 l oxygen/min, or increased work of breathing. This notification allows the ART team to monitor these patients for signs of respiratory deterioration and facilitate early intubation as needed. In addition, it ensures that ART is aware of patients with difficult airways or other relevant comorbidities so that they may better prepare for these issues. In many institutions, the ART is composed of providers who are simultaneously caring patients in the ICU and OR. Their ART duties are in addition to their primary roles as intensivists, anesthesiologists, emergency medicine physicians, or surgeons. It is generally not feasible for the ART to assess every patient who presents with general viral or flu-like symptoms without evidence of active respiratory distress; rather, the ART should be consulted when there are early signs of respiratory insufficiency (Fig*.* 1). When consulting the airway team, the primary team should provide a concise and relevant medical report. This should include any prior documented intubations, most recent echocardiography results, comorbidities, and recent clinical course including hemodynamic and/or respiratory instability, oxygen requirements, and objective work of breathing (Fig. 2).

In a recent letter, Cheung et al. provided recommendations for airway management of patients with COVID19 based on their experience in Hong Kong which we have adapted and expanded upon in the following sections [7].

## Minimize non-invasive positive pressure ventilation and high-flow nasal cannula

Non-invasive positive pressure ventilation and high-flow nasal cannula (HFNC) carry a risk of COVID-19 aerosolization and transmission, and their use should be implemented with discretion. While intubation creates a situation of acute high-risk exposure for health care providers for the duration of the intubation, the use of non-invasive ventilation (NIV) with BiPAP, CPAP, or HFNC compounds this risk through continuous open circuit viral aerosolization. If the patient is not in a negative pressure room, this aerosolized viral shedding can extend beyond the room, contaminating the hallways and beyond. Therefore, use of NIV for COVID-19 patients should be minimized and limited to negative pressure rooms if required. For this same reason, some literature recommends that all patients requiring oxygen flows greater than 6 L/min be cared for in a negative pressure room [7]. Despite concerns for viral transmission, NIV may be required to achieve adequate preoxygenation for safe intubation of a severely hypoxemic patient. The provider should make every attempt to minimize air leaks by ensuring a tight seal. The benefits of using NIV must be weighed against the risks of transmission to healthcare workers even in negative pressure rooms and the availability of other resources such as ventilators, specialized personnel, negative pressure rooms, and ICU beds.

The decision to intubate serves to provide necessary oxygen support prior to accumulation of oxygen debt, decrease the contribution of patient self-inflicted lung injury (P-SILI) to severe or worsening ARDS, and to preempt the need for a high-risk emergent intubation. Although decreased aerosolization is one consideration, it is important to recognize that early intubation and institution of lung protective ventilation may decrease the risk of progression to severe acute respiratory distress syndrome (ARDS) in COVID-19 patients. Use of intubation as a rescue therapy rather than as a proactive treatment for a mounting oxygen debt in patients with moderate to severe COVID-19 infections may have contributed to increased mortality early in the epidemic [13]. Further, spontaneously breathing patients with COVID-19 have a high respiratory drive that can result in injurious transpulmonary pressure swings and self-inflicted worsening of ARDS [14].

The ART must consider elective intubation for proactive management of ARDS and viral containment in any patient with increasing oxygen requirement above 6 L/min, worsening PaO2/FiO2 ratio, or increased work of breathing or respiratory rate or for whom the need for NIV or HFNC is being considered [15]. Whether intubation is appropriate in patients meeting the above decompensating criteria must be a clinical case-by-case decision; however, it is imperative that intubation not be a consideration reserved for those in extremis. The decision for early intubation will need to take into account the availability of resources such as ventilators, specialized personnel, and ICU beds. Early arterial line placement may be beneficial in the patient with worsening respiratory status to monitor gas exchange and manage hemodynamics during intubation.

## Intubating the COVID-19 patient: failing to prepare is preparing to fail

In preparation for intubation, patients must have intravenous access and at minimum the basic physiologic monitors recommended by the American Society of Anesthesiologists. This includes continuous pulse oximetry, ECG, and blood pressure monitoring. The ART will need working suction, availability of appropriate difficult airway equipment including laryngeal mask airways (LMA), PEEP valve, gum elastic bougies, video laryngoscope, colorimetric capnography or, ideally, waveform capnography, and a ventilator at bedside. All necessary equipment and medications should be prepared prior to entry into the room of a patient with COVID-19 infection so as to minimize the duration of possible exposure.

The team should ensure that a high minimum efficiency reporting value (MERV) rated filter, such as a high efficiency particulate air (HEPA) filter, is placed on the ventilator circuit directly at the site of connection with the endotracheal tube prior to use [16]. Based on patient comorbidities and potential hemodynamic instability during the intubation process, appropriate vasopressors should be available and in line prior to intubation.

There are several special procedure-related considerations for intubation that the team should bear in mind. To minimize exposure of healthcare providers during intubation, patients with suspected or confirmed COVID-19 should be roomed in negative pressure suites with full airborne precautions. The choice of neuromuscular blocking agents for these patients remains a topic of debate. Cheung et al. [7] recommend a rapid sequence intubation approach using a high dose of nondepolarizing agent, such as rocuronium, rather than succinylcholine. The longer duration of action of rocuronium prevents aerosolization via patient coughing in the event of multiple attempts at intubation, whereas succinylcholine has a duration of effect lasting only 3–5 min. An increased dose of rocuronium (greater than or equal to 1.2 mg/kg) reduces time to drug onset, which reduces the risk of patient coughing during intubation. Alternatively, succinylcholine given its rapid onset and recovery time may be preferred in the absence of contraindications such as long-term immobility, family history of malignant hyperthermia, certain neuromuscular disorders, and marked hyperkalemia. Use of intravenous lidocaine (1.5 mg/kg) and avoidance of fentanyl are additional strategies which may help prevent coughing. Lidocaine should be used with caution and should be avoided in hemodynamically unstable patients. Awake fiberoptic intubation should be minimized in COVID-19-positive patients as coughing is common with this procedure, conferring risk of infection to those involved. Atomization of local anesthetic for airway topicalization, as is required for these intubations, also risks aerosolizing patient sputum [15].

Bag-mask ventilation should be avoided in COVID-19 patients when possible. Prior studies have found that manual ventilation before intubation was associated with an increased risk of SARS transmission [8] and poses a similar risk for COVID-19 transmission. High-quality preoxygenation with 100% inspired oxygen for 5–10 min is important to optimize patients prior to airway management. Some centers recommend using NIV for preoxygenation based on studies of non-COVID-19 patients with acute hypoxemic respiratory failure [17]. If this approach is used, the ventilator must be switched off between preoxygenation and intubation to decrease aerosolization. Our institution does not use this approach.

If post-induction ventilation is required prior to intubation, some experts recommend placing an LMA immediately after induction for potentially lower risk of aerosolization. If bag-mask ventilation is required, low tidal volumes should be used and all precautions should be taken to avoid leaks. Air flows should be switched off during laryngoscopy. If a difficult airway is anticipated, or if the patient shows signs of tenuous oxygenation and a risk of rapid desaturation, a surgical airway team should be prepared at bedside. Routine use of video laryngoscopy has been suggested to provide additional distance between the intubating clinician and the airway. We also recommend a low threshold to escalate to a surgical airway in order to avoid repeated instrumentation of a difficult airway in COVID-19 patients and the subsequent precipitous emergent surgical airway. All clinicians involved in airway management should be clearly informed of the patient’s COVID-19 confirmed or presumptive diagnosis, and the most experienced provider should perform the intubation. To further minimize exposure, the number of providers present in the room during intubation should be limited to only those who are essential.

Given the extremely high risk of viral transmission to all involved in the care of an arresting COVID-19 patient actively receiving chest compressions, it is crucial that all providers properly don PPE prior to attempting intubation or bag-mask ventilation. Although PPE should be made available, if a circumstance arises where it is not possible to don PPE, LMA placement rather than endotracheal intubation should be considered. Intubation may be attempted in a more controlled setting after return of spontaneous circulation (ROSC).

## Post-intubation considerations

Following intubation, providers should minimize the time to inflation of the ETT cuff and connection to the ventilator circuit. Any equipment that was in contact with the airway such as laryngoscope blades and masks should be immediately disposed of or contained within a plastic bag for decontamination. While disposal is preferred from an infectious risk, containment may be appropriate to conserve supplies for repeat use on the same patient. Any contaminated PPE should be removed as soon as possible.

**Fig. 3:**
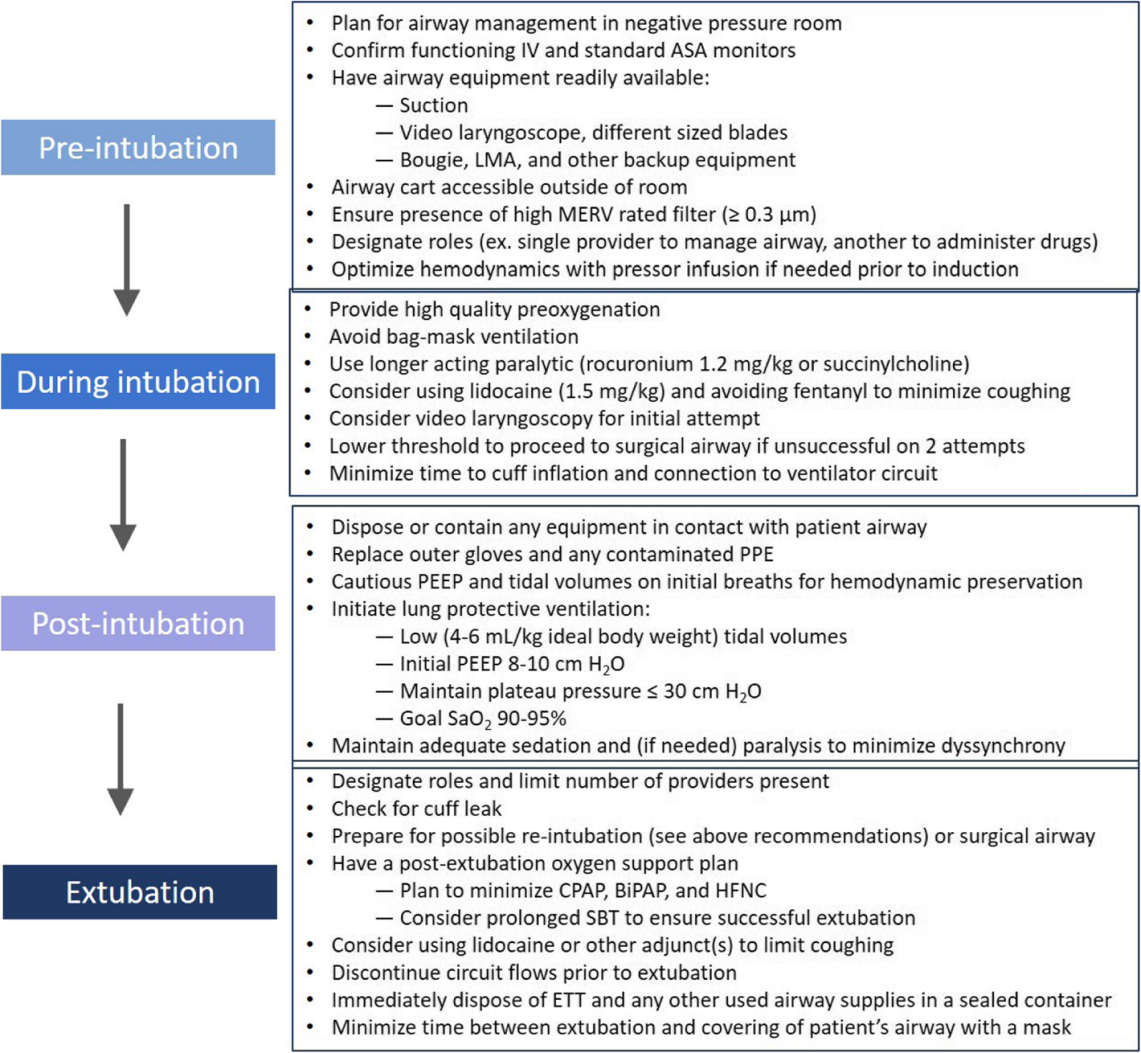
Summary of current recommendations for airway management of suspected or confirmed COVID-19 patients

Protective ventilation should only be initiated after hemodynamic stabilization in order to minimize the hemodynamic effects associated with high PEEP and respiratory rates, such as right heart dysfunction and decreases in preload. Data on the ideal ventilation parameters specific to COVID-19 patients is lacking. Because many (up to 67% by recent report [18]) of critically ill COVID-19 patients may develop ARDS, clinicians should consider lung ventilation strategies that have been established for the management of ARDS (Fig. 3). Tidal volume and PEEP may then be titrated based on each patient’s lung compliance.

It is also important to maintain appropriate levels of sedation and, if necessary, paralysis to limit patient-ventilator dyssynchrony. Adequate paralysis should be confirmed prior to performing any recruitment maneuvers. Dyssynchrony can lead to inadequate ventilation, lung injury, hemodynamic instability, and increased risk of exposure if healthcare workers are needing to repeatedly enter patient rooms for ventilator adjustments. Proning, nitric oxide, and veno-venous ECMO are additional measures that can be considered for refractory hypoxemia. While there is conflicting evidence on potential benefits of nitric oxide in patients with severe ARDS, the use of nitric oxide in COVID-19 patients is being actively studied.

## Extubating the COVID-19 patient

Extubation poses another significant risk of COVID-19 transmission. With less direct mucous membrane contact, but potentially higher risk of cough aerosolization, extubation should be performed with caution. Additionally, findings of continued viral shedding after the resolution of acute illness highlight the important fact that clinical improvement does not equate with non-infectiousness [11].

It is recommended to strategically but safely limit the number of people present for extubation. Every extubation carries a risk of re-intubation, further elevating infectious risk to healthcare providers for the reasons previously mentioned. The ART should be immediately available, and the patient should be reintubated early if there is evidence of respiratory failure after extubation. Since NIV and HFNC pose risk in these patients, a thorough post-extubation oxygen support plan should be established prior to extubation. These patients should not be extubated to room air, as there are few risks to low flow oxygen support, while the risks of respiratory failure and subsequent reintubation are high. Aerosolizing nebulizer treatments post-extubation should be ideally avoided or at least minimized in favor of metered dose inhalers if necessary. The benefits of using NIV post-extubation should be weighed against the risk of transmission to health care workers. In such scenarios, a tracheostomy rather than a trial extubation should also be considered, especially if the patient was a difficult intubation. Tube exchangers may also be considered for patients who undergo a trial extubation, and reintubation is anticipated to be challenging.

Of note, given the prolonged intubation and frequent use of intermittent proning in this patient population, these patients may be at higher risk for airway and vocal cord edema. We recommend routinely checking for a cuff leak prior to extubation.

## How long does COVID-19 stay behind?

As we consider the infectious risks of airway management in COVID-19 patients, it is helpful to contextualize risk of transmission with the length of time a contaminated surface remains a threat. van Doremalen et al. found that COVID-19 is viable in aerosols for at least 3 h, on copper for 4 h, and on cardboard for 24 h. COVID-19 is most stable on plastic and stainless steel, two of the more common surfaces found in the hospital setting. COVID-19 was found to be viable and infectious on plastic and stainless steel for 72 h after exposure. This must be considered when addressing the plan for high-risk COVID-19 intubations and the plan for post-intubation decontamination.

## Designation of a COVID-19 unit

The availability of negative pressure rooms, mechanical ventilators, and appropriate staffing may be limited as the pandemic continues, and clinical judgment for maximizing their use will be required. We recommend advanced planning for this likely scenario. At our institution, we have 94 negative pressure rooms, 18 of which are in an ICU. If the pandemic need surpasses 94, we have diverted all pediatric cases to the nearby Children’s Hospital in order to convert our pediatric ICU (PICU) into a dedicated COVID-19 unit and our post-anesthesia care units (PACUs) into an over 90-bed ICU.

The availability of ventilators is another grave consideration. We recommend that each institution consider canceling elective surgical cases as the pandemic grows. Although a difficult decision, canceling elective surgical cases will decrease transmission between the community, health care providers, and the in-hospital populace. Furthermore, operating room ventilators may have to be utilized for care of patients with respiratory failure from COVID-19 if the number of available ICU ventilators is exceeded. If the institution is unable to create a COVID-19 unit, then the operating rooms themselves may be an alternative to ICU beds.

## Conclusion

As the pandemic continues to spread, it is our hope that the same interconnectedness that allowed the spread of COVID-19 will also allow for rapid dispersal of evidence-based management recommendations. The most recent recommendations include close monitoring of respiratory status for early signs of failure, cautious use of NIV and HFNC and consideration of early intubation, use of video laryngoscopy, and minimizing coughing and viral aerosolization during induction with medications such as lidocaine and high dose rocuronium. We also emphasize efforts to reduce infectious risk such as by limiting the providers caring for COVID-19 patients to a select, experienced group of clinicians and ensuring proper availability, donning, and doffing of airborne precaution PPE. Our approach has been effective in caring for our patients and our healthcare workers; with a minimum follow-up of 30 days, our critically ill intubated COVID-19-positive ARDS patients have had a mortality rate of 16.7%, the majority successfully extubated and discharged from the ICU [19]. A well-planned and efficiently executed strategy centered on caring for our patients while protecting our staff will continue to be critical in ensuring optimal care and containing this pandemic.

## Data Availability

Not applicable.

## Abbreviations

COVID-19: Coronavirus disease 2019
NIV: Non-invasive ventilation
HFNC: High-flow nasal cannula
BiPAP: Bilevel positive airway pressure
CPAP: Continuous positive airway pressure
SARS: Severe acute respiratory syndrome; a viral respiratory illness due to SARS-associated coronavirus (SARS-CoV)
ARDS: Acute respiratory distress syndrome
P-SILI: Patient self-inflicted lung injury
CRNA: Certified registered nurse anesthetist
AA: Anesthesiologist assistant
ICU: Intensive care unit
PICU: Pediatric ICU
PACU: Post-anesthesia care unit; the postoperative recovery unit
OR: Operating room
Code status: The formalized expression of a patient’s wishes for level of medical intervention in the event of cardiac or respiratory failure
